# ‘What Do I Know? Should I Participate?’ Considerations on Participation in HIV Related Research among HIV Infected Adults in Bangalore, South India

**DOI:** 10.1371/journal.pone.0053054

**Published:** 2013-02-27

**Authors:** Rashmi J. Rodrigues, Jimmy Antony, Shubha Krishnamurthy, Anita Shet, Ayesha De Costa

**Affiliations:** 1 Division of Global Health, Karolinska Institute, Stockholm, Sweden; 2 St. John's National Academy of Health Sciences, Bangalore, India; University of Ottawa, Canada

## Abstract

**Background:**

India has the highest number of HIV infected persons in the world after South Africa. Much HIV related behavioral, clinical and laboratory based research is ongoing in India. Yet little is known on Indian HIV patients' knowledge of research, their processes of decision making and motives for participation. We aimed to explore these areas among HIV infected individuals to understand their reasons for participating in research.

**Methodology/Principal Findings:**

This is a cross sectional survey among 173 HIV infected adults at a tertiary level hospital in Bangalore, India, done between October 2010 and January 2011. A pre-tested questionnaire was administered to the participants by trained research assistants to assess their knowledge regarding research, willingness to participate, decision making and determinants of participation. Participants were presented with five hypothetical HIV research studies. Each study had a different level of intervention and time commitment. Of respondents, 103(60%), said that research meant ‘to discover something new’ and 138(80%) were willing to participate in research. A third of the respondents were unaware of their right to refuse participation. Willingness to participate in research varied with level of intervention. It was the lowest for the hypothetical study involving sensitive questions followed by the hypothetical drug trial; and was the highest for the hypothetical cross sectional questionnaire based study (p<0.0015). Individual health benefits and altruism were the primary motives for participation in research and indicate the presence of therapeutic misconception. Women were less likely to make autonomous decisions for participation in interventional studies.

**Conclusions/Significance:**

Despite a majority willing to participate, over a third of respondents did not have any knowledge of research or the voluntary nature of participation. This has ethical implications. Researchers need to focus on enabling potential research participants understand the concepts of research, promote autonomous decisions, especially by women and restrict therapeutic misconception.

## Introduction

Studies on the contextualization of ethical processes, particularly informed consent [1], have been reported in literature from several low income settings [2]–[4]. Some of these studies have attempted to understand the influence of knowledge regarding research, poverty, illiteracy and culture on research participation and the ethical dilemmas arising thereof [5].

Ethical challenges in HIV research are complex because of the tonicity of the condition, socioeconomic vulnerability and stigma experienced by the infected person [6]. Hopes of obtaining a cure, prolonging life or just accessing treatment influence a larger number of HIV positive individuals to participate in HIV research compared to individuals suffering from other chronic diseases [7]. Studies have reported that 14% of HIV-infected individuals on treatment participate in clinical trials in comparison to 1.5–4% on cancer treatment. This impressive participation of HIV positive individuals in research has been responsible for the success of several advances in the management of HIV [8]. A study from South India reported high willingness to participate in HIV vaccine trials motivated by the desire to be protected from HIV [9].

Given that there are 2.5 million HIV infected individuals in India [10], an increasing number of research studies are now taking place in the Indian subcontinent [5]. Consequently, there have been calls for improving our understanding of the various ethical and societal concerns related to HIV research in the country [11]. Existing literature addressing the ethical issues around HIV in India has dealt with HIV testing [12], stigma [13], disclosure, drug trials [14] and in the recent years, preparedness for vaccine trials [15]. Guidelines that address operations research in HIV/AIDS have been developed by the National AIDS control program and the Indian Council of Medical Research [16], [17]. These guidelines ensure that universal ethical values are adapted to suit the local socio-cultural context.

Ethical guidelines require that potential participants be informed regarding the purpose of research, the type of study, its duration and the risks and benefits of participation prior to obtaining consent. This ensures that the four principles of informed consent, i.e.; autonomy, voluntariness, non-maleficence and justice are adhered to in the conduct of research [16]. Informed consent assumes that research participants have some knowledge of the basic concepts of research. However, many low and some middle-income settings, (including India) have relatively high levels of illiteracy making it likely for potential research participants in such settings to have little knowledge of the basic concepts of research. Given this scenario it was thought necessary to study HIV patients' understanding of the concept of research, their willingness to participate in research, their decision making process and motives for participation in research in the Indian context. In addition the associations between these variables and the influence of the level of research intervention on willingness to participate in research and decision making for participation were also studied.

## Methods

This cross sectional survey was conducted between October 2010 and January 2011 at the Infectious Disease Clinic, St. John's National Academy of Health Sciences, Bangalore, South India. This is a large tertiary care hospital clinic that provides routine care and treatment to approximately 2000 HIV-infected individuals from within the province of Karnataka and adjoining provinces. The hospital provides outpatient care and basic follow-up investigations at no cost to all HIV infected individuals irrespective of income, through a public private partnership.

HIV positive patients above the age of 18 years who attended the infectious disease clinic for routine follow up were invited to participate in the study. An interviewer administered, semi-structured, questionnaire was used in the study. The questionnaire was pre-tested for feasibility and participant comprehension. The questionnaire was then suitably modified based on participant and interviewer feedback. Trained research assistants, not involved in routine patient care, spent three hours a day, thrice a week at the clinic, recruiting and administering the questionnaire to participants. The questionnaire was administered to participants in the local language over 20 minutes.

The questionnaire elicited participants' understanding of the concept of research, their motives for participation and their decision making processes for participation. The participants were presented with five hypothetical HIV research study scenarios. Each study scenario involved a different level of intervention and required participants to be committed to the study for varying lengths of time. These study scenarios are described in *table* *1* and included, (i) a drug trial that involved blood draws and quarterly follow-ups for a period of two years (ii) a longitudinal observational cohort that studied the progress of HIV in the patient (iii) a single blood draw to study viral loads (iv) a cross sectional questionnaire study that assessed quality of life and (v) a cross sectional study with sensitive questions particularly related to sexuality. For each hypothetical study scenario, participants were asked about their willingness to participate, their motives and their decision making processes for participation. However, after the assessment of knowledge regarding research and prior to the assessment of willingness to participate in research all participants were briefly educated regarding the concept of research and their ability to refuse research participation. The questionnaire also elicited socio-demographic information for each participant.

**Table 1:** Hypothetical HIV research scenarios and questions.

I	HIV research scenario description
**1**	Consider that a group of researchers want to study a new medicine to improve survival rates in HIV patients. The study is for a period of 2 years and will require you to visit the hospital regularly for follow-ups. You will also have to give some blood upto 20ml for laboratory tests a few times during the visits. The investigations will be free. If the researchers request you to participate in the study would you be willing to participate? *Answer: 1. Yes 2. No*
**2**	A group of researchers want to study the signs and symptoms of HIV. They will observe HIV positive people lifelong to see how the disease progresses and what treatment works. You do not have to do anything more than visit the clinic regularly for appraisal or medications as you are doing now. The information about your health collected at these visits will be analysed for the purpose of this study. Would you be willing to participate? *Answer: 1. Yes 2. No*
**3**	A group of researchers want to study the type and amount of virus you have in your blood. It requires that you give approximately 20 ml of blood, once. The investigation is free. Would you be willing to participate in this study? *Answer: 1. Yes 2. No*
**4**	A group of researchers want to study the quality of life of people with HIV. They approach you and request you to participate. The questionnaire will take approximately 20 minutes of your time. Will you be willing to participate? *Answer: 1. Yes 2. No*
**5**	If instead of the quality of life in the above scenario researchers request you to participate in a study that asked you sensitive and personal information that would enable them to understand more about how the disease spreads, would you be willing to participate? *Answer: 1. Yes 2. No*

II	Questions related to each scenario
**1**	A. If willing to participate why?
	B. If not willing to participate why?
**2**	Who would make the decision for you participate/not participate in this study?
**3**	Would you have a say in making the decision?
**4**	How long will you require to make your decision?
**5**	Why would you require time to make your decision?
**6**	Would you need to consult anybody to make your decision?

Data was analysed with SPSS version 16. Frequencies, mean, median and standard deviation were used to describe quantitative variables. In the case of open ended questions, answers were recorded verbatim, and subsequently categorized to enable frequency counts and their presentation as descriptive variables. Knowledge of the concept of research, willingness to participate and decision making for participation were considered as ‘outcome’ variables. Socio-demographic characteristics were studied against these outcomes and associations expressed as odds ratios with 95% confidence limits. The Chi-square test, t-test and stepwise logistic regression models (forward LR) were used to study associations. Cochran's Q was used to identify intra-individual variability in willingness to participate and decision making based on the different types of hypothetical study scenarios. To further attribute intra-individual variability between responses between any two types of hypothetical studies the McNemar's test was used and the significance (p value) subjected to Bonferroni's correction.

The study and its informed consent process was approved by the Institutional Ethics Review Board at St. John's National Academy of Health Sciences, Bangalore, Karnataka, India. Verbal consent to participate in the study was obtained from potential participants and documented by the person administering consent in the presence of a witness. This process was also endorsed by the witness.

## Results

Overall, 239 patients were approached, of whom 173 consented to participate. Thirty five percent of those recruited were women reflecting the proportion of women patients seen in the clinic. Time constraint was the most common reason for nonparticipation. The demographic characteristics of the 173 respondents are described in *table* *2*.

**Table 2:** Socio-demographic characteristics of participants.

Characteristics (N = 173)	Total	Female	Male
**Sex**	173	61(36%)	112 (64%)
**Age(mean**+**sd**)	39±8.8	35.24±8.2	41.71±8.2
**Residence:**			
Urban	143(83%)	51 (36%)	92 (64%)
Rural	30(17%)	10 (33%)	20 (67%)
**Education:**			
Nil	15 (8%)	8 (53%)	7 (47%)^*^
≤7yrs	34 (20%)	16 (47%)	18 (53%)^*^
>7yrs	124 (72%)	37 (30%)	87 (70%)
**Family type**			
Lives alone/Nuclear	37 (21%)	47 (35%)	89 (65%)
Others#	136 (79%)	14 (39%)	23 (62%)
**Employed**			
Yes	144 (83%)	30 (22%)	104 (77%)
No	39 (17%)	31 (80%)	8 (20%)
**Monthly Family Income:**			
Median (IQ range) in USD$	140 (80–240)	140 (80–220)	150 (90–256)
<150USD	78 (45%)	31 (40%)	47 (60%)
≥150USD	95 (55%)	30 (30%)	65 (70%)
**Prior participation in research**			
No	107 (62%)	41(38%)	66(66%)
Yes	66 (38%)	20(30%)	46(68%)
**CD4 count (N = 170)**			
**</ = 200**	50 (71%)	20 (29%)	70 (41%)
**201–350**	19 (33%)	14 (42%)	33 (19%)
**>350**	43 (64%)	24 (36%)	67 (39%)
**HIV stage**			
Stage I	131 (76%)	50(38%)	81(62%)
Others	42 (24%)	11(26%)	31(74%)

$ 1USD approximately  = 50INR, ^*^categories combined for logistic regression analysis.

# Joint, three generation or extended family.

### What is research?

Of the 173 participants, 103 (60%) had some knowledge of the concept of research. Of these for 93%, research meant ‘finding something new’. Women (adjusted OR = 0.39, CI: 0.16–0.83), those with ≤7 years of education (adjusted OR = 0.13, CI: 0.03–0.31), those unemployed (adjusted OR = 0.34, CI: 0.14–0.94) and those who had never participated in research (adjusted OR = 0.40, CI = 0.20-0.98) were significantly less likely to have an understanding of the concept of research (*derived from* *table* *3*).

**Table 3:** Association of knowledge regarding research with demographic variables.

	Bivariate		
	P value	OR	Adjusted OR
Age	0.031		
**Male**	**<0.001**	**4.13 (2.134–7.99)**	**2.65 (1.12–6.28)** *
**>7years education**	**<0.001**	**8.50 (3.96–18.24)**	**7.42 (3.26–16.90)** *
Joint/extended family	0.128	0.57 (0.27–1.18)	
Urban	0.446	1.36 (0.62–3.00)	
Employed	**<0.001**	**4.09 (1.92–8.72)**	**2.89 (1.07–7.81)** *
Income ≥150USD	0.448	1.27 (0.69–2.33)	
CD4 Count	0.393		
HIV stage >1	0.679	0.39 (0.34–1.39)	
**Prior Research participation**	**0.002**	**2.83 (1.45–5.53)**	**2.250 (1.024–4.944)** *

* Variables included in the final step of the final step of the logistic regression model.

#### Risk perception

Of the 103 participants who had an understanding of the concept of research, 62% perceived no risk, 25% recognized some risk (i.e. adverse drug reactions, time lost, human rights violations, privacy and confidentiality issues) and 13% did not know of any risk associated with research participation.

#### Who benefits from research?

Patients and their families (55%), humanity (37%) or the medical fraternity (15%) were considered beneficiaries of research.

The concept of research was explained in a standardized format to those participants who did not know about research, prior to administering the following questions.

#### Can one refuse participation?

Of the 173 participants, 63 (36%) were unaware that they could refuse to participate in research. Of these 37(59%) considered it was in their best interests to participate, 16 (25%) cited altruistic compulsions for participation and 15 (23%) perceived participation as obligatory. It was more likely for participants who were urban (adjusted OR = 2.95, CI: 1.32–6.61) or employed (adjusted OR = 2.49, CI = 1.16–5.29) to know that they could refuse to participate in research (*table* *4*).

**Table 4:** Association between perception that participation in research was compulsory with demographic variables.

	Bivariate		
	P value	OR	Adjusted OR
Age	0.389		
Male	0.027	1.06****(0.55–2.06)	
>7years education	0.018	2.26****(1.14–4.45)	
Joint/extended family	0.711	0.87****(0.35–1.84)	
**Urban**	**0.007**	**2.94 (1.32–6.58)**	**2.95 (1.32–6.61)** *
**Employed**	**0.017**	**2.39 (1.15–4.94)**	**2.48 (1.16–5.29)** *
Income ≥150USD	0.872	0.95****(0.51–1.78)	
CD4 count	0.562		
HIV stage>1	0.12	0.57****(0.28–1.16)	
Previous Research Participation	0.284	1.43****(0.74–2.71)	

* Variables included in the final step of the logistic regression model.

#### Are you willing to participate?

Of the 173 participants, 138 (80%) were willing to participate in research. Of those unwilling, 12 (34%) were uncomfortable with the idea, 8 (23%) cited privacy concerns and 6 (17%) reported time constraints as reasons for refusal. Participants who had previously participated in research were more likely to be willing to participate in research (adjusted OR = 8.16, CI = 2.38–27.98) *(* *table* *5*).

**Table 5:** Association between willingness to participate in research with demographic variables.

	Bivariate		
	P value	OR	Adjusted OR
Age	0.928		
Male	0.794	1.11 (0.51–2.39)	
>7years education	0.977	1.02 (0.45–2.31)	
Joint/extended family	0.823	1.11 (0.44–2.79)	
Urban	0.125	0.39 (0.11–1.35)	
Employed	0.078	0.38 (0.13–1.15)	
Income ≥150USD	0.221	1.59 (0.75–3.35)	
CD4 count	0.275		
HIV stage>1	0.509	1.36 (0.55–3.39)	
**Previous Research Participation**	**<0.001**	**6.32 (2.12–18.88)**	**8.158 (2.379–27.979)** *

* Variables included in the final step of the logistic regression model.

### Hypothetical HIV research study scenarios: participation and decision making

#### Will you participate?

**Figure 1:**
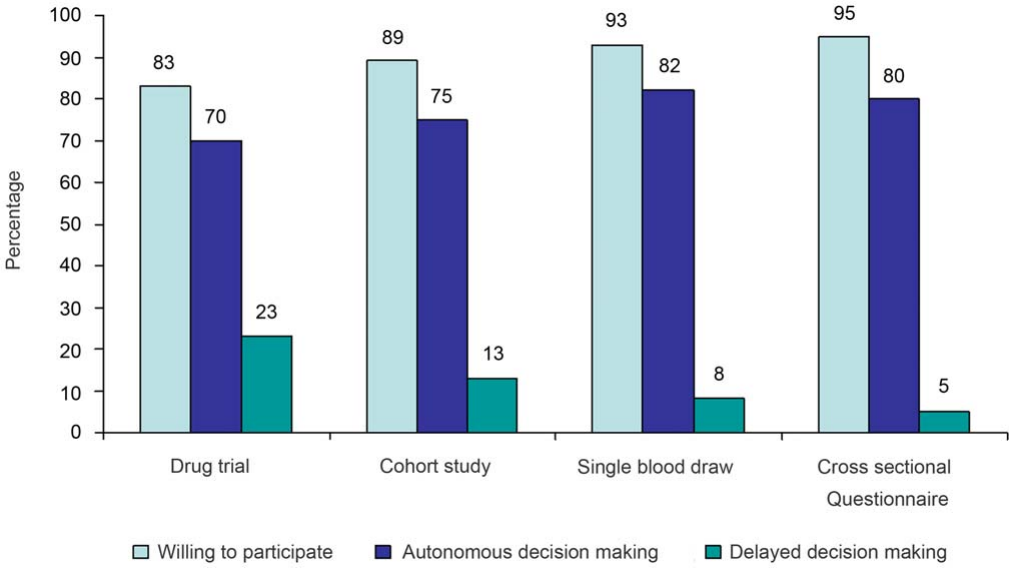
Research participation and decision making in hypothetical HIV research studies. (n = 138).

Five hypothetical HIV research studies were proposed to the 138 participants willing to participate in research. The results are shown in *Figure* *1*. Willingness to participate was the least where the research scenario involved answering sensitive questions (81% – not shown in the figure).

Participants were more willing to participate in a study involving a single blood draw or a cross sectional questionnaire in comparison to a drug trial or an observational cohort (p<0.01). Eighty nine (64%) of the participants were willing to participate in any type of study proposed. Self-interest was the main motivation for over two thirds of the participants for all study types, except the cross sectional survey, where 28% stated that the opportunity to ‘share their experiences’ was a motivation. Half of all participants reported altruism as a motive for participation in any of the studies. Time constraint was the reason cited for refusing to participate in the hypothetical drug trial and observational cohort study. Men (adjusted OR = 2.60, CI = 1.34–5.09) and those in clinical stage I of the disease (adjusted OR = 2.93, CI = 1.34–6.46) were more likely to have altruistic motives for participation in research.

#### Decision making (Figure 1)

Of the 138 participants willing to participate in research, 65%–72% (based on the type of study) would make the decision to participate by themselves (p<0.05). Of those willing to participate in each of the hypothetical studies, women were less likely to make autonomous decisions to participate in a hypothetical drug trial, an observational cohort or a single blood draw study (p<0.05). A third to 40% of participants were unable to make their decision on the same day that they were invited to participate. The primary reasons identified for delayed decision making (i.e.; >1 day for decision making) were the need to understand the research study or consult a spouse/family. Women were significantly less likely to make their decision to participate on the same day for a hypothetical drug trial, an observational cohort or a single blood draw study (p<0.05).

## Discussion

Stigma, income poverty, lack of formal education, substance abuse and unemployment, especially in low and middle income settings imply that a clear understanding of research and its potential risks and benefits could be problematic particularly in these settings [6]. This is also evidenced by the results from our study where vulnerable groups including women, those with ≤7 years of education or those unemployed were significantly less likely to have an understanding of research. Those with >7 years of formal education were aware that they could refuse participation in research. Willingness to participate in research was not dependent on knowledge in our study. However, prior participation in research was associated with a greater willingness to participate in research and a better knowledge of research.

Our results indicated that 40% of the respondents did not understand the concept of research. Similar reports of limited knowledge regarding research were also made by a study in KwaZulu-Natal, South Africa [18]. However, despite their limited understanding, participants in the South African study considered research to be an “enquiry into knowledge”, which was similar to the finding in our study. Reports of similar findings are also available from Sri Lanka [19]. Despite most patients being educated in our study the limited exposure to the concept of research in formal education or social discourse probably contributed to the limited knowledge regarding research.

Knowledge regarding research is known to influence research participation both positively and negatively [5], [20]. Studies from the USA and United Kingdom report that the perception of researchers exploiting participants as a barrier to research participation. These studies recommend educating patients to improve research participation [5], [20], [21]. On the contrary in our setting, given the limited knowledge regarding research and the paternalistic nature of the doctor-patient relationship, few considered such exploitation a possibility. Therefore unlike high-income settings, in our setting, researchers should focus on educating potential participants regarding the concept of research, participant rights and the pros and cons of participation. Information should be communicated and interpreted culturally [22]. This would ensure that potential participants are not overwhelmed by the complexity of information provided but are able to understand it [22]. Reports of research participants confronting researchers regarding published information despite consenting are available in literature [23]. The researcher considers the possibility that the participant had consented to a process the product of which she could not conceive at the time of consent [23]. Similarly in our setting several participants probably did not know what they were consenting to given their limited knowledge regarding research and their desire for better health. It is possible for these participants to acquire knowledge and question their decisions in retrospect, which on the one hand could help confirm decisions to participate while on the other hand, might result in participants having doubts around their decision along with feelings of exploitation. It is therefore a better alternative for researchers to exclude from research studies those participants that fail to understand the concept of research, its risks and benefits, and the voluntary nature of participation, even after they are explained to them. Such efforts will ensure that the principle of non-maleficence is not diluted by cultural and contextual relativism.

One third of all respondents in our study were not aware that research participation was voluntary. Research participants tend to confuse research with clinical care, when research is conducted in their routine healthcare setting, particularly in settings where paternalistic attitudes govern doctor patient relationships [24]. In such settings potential participants may consider an invitation to enroll in research as a professional recommendation by their physician [25]. A study on participation in research and access to experimental treatments in the United States reported that the request to participate in research by their primary care physician was the strongest predictor of participation in research by HIV infected patients [26]. Reports of potential participants' perception of being obliged to participate in research in a hospital setting are also available from South Africa. Eighty eight percent of the participants in the South African study felt compelled to participate in research implying that participation was less than voluntary [27]. Studies in the Indian setting indicate that general practitioners/family physicians play an important role in the healthcare decision making process [5]. By contrast in less paternalistic settings like USA, only two percent of respondents felt participation in research was compulsory [28]. The finding in our study that 40% of the participants did not know what research meant and a third did not recognize that they could refuse participation implies that for a third of the patients, research participation could be possibly involuntary. Given this scenario, it is likely that a significant proportion of research participants in similar low–middle income contexts are not truly informed despite researchers' theoretical adherence to universal ethical principles, as participants probably do not fully comprehend the implications of participation. The issue may be addressed through pre-recruitment counseling prior to consenting potential participants for research [6]. Such counseling should focus on voluntariness of research participation and availability of uncompromised care irrespective of the decision to participate in research [6]. Further researchers should assess participant preparedness for research after pre-recruitment counseling [6]. *Willingness to participate*: This was influenced by the type of hypothetical study and was the least (yet relatively high) for studies containing sensitive questions related to sexual behavior. Most of those who refused to participate in studies involving such sensitive questions were unemployed women, possibly because the question addressed sexual behavior, the discussion of which may have been considered culturally a taboo [29]. Studies on the ethics of reproductive health in South India report that the discussion of reproductive health issues with strangers is considered inauspicious and could be an issue for the family [23].

Willingness to participate in the hypothetical HIV drug trial was higher than that reported for a hypothetical non-HIV drug trial (83% Vs. 30%) in South India [30]. Potential reasons for the higher participation by HIV patients in research could include access to care and optimism about a cure for the condition. Unlike other drug trials, uncertainties around taking a new drug did not feature significantly as a barrier to participate in research in our study [5]. The hope of a cure and an opportunity for better treatment and follow-up were reasons reported for willingness to participate in the hypothetical drug trial. In such a situation the ethical principle of justice is compromised [31]. To prevent this, researchers should justify involving a specific population in research. Researchers need to demonstrate that the benefit to these populations overrides the risk of involving them in research [31]. Further, if the new treatment is successful, researchers should have a realistic plan to ensure its availability in the study setting [31].

We found the willingness to participate in a hypothetical observational cohort to be higher than in a hypothetical drug trial. This may have been due to respondents' perception that an observational cohort study was similar to routine care. Research was considered as an integral service of the hospital by participants in a South African study [27]. Narrative reports of researchers mistaken for gynecologists experienced in treating infertility are available from South India [23]. Such perceptions compromise informed consent.

Completion of the study at a single visit in our study, further increased the willingness to participate. Study designs that minimally interfere with routine life have been found to encourage participation. As in earlier reports [5], time constraints were expressed as a barrier to participation in drug trials in our study.

### Motives for participation

Previous studies have reported self-interest and altruism to be primary reasons for participation in research. This was also seen in our study. Patients in the Indian setting have been found to participate more willingly in research when they believed that they would benefit in terms of good health and protection from disease. Studies have also shown that research participants do not readily differentiate between research and medical care creating a therapeutic misconception [32]. The self-interest motivating some patients in our study could imply a degree of therapeutic misconception. Altruistic attitudes have been identified to influence research participation in many settings, but are more likely so in HIV research, where respondents hope that others like them will benefit [33].

An important motive for participation in a cross sectional questionnaire study that we identified was the opportunity to share experiences with the researcher. Another study has also reported similar findings [34]. Studies also report that research participants like to communicate in ways that embrace both emotional and medical concerns [30]. Researchers have been considered as counselors for adoption, family conicts, stigma and discrimination at a reproductive health clinic in South India both by patients and physicians. In the south Indian study, research provided patients with an opportunity to share their distress around societal reaction to their infertility. This would not be possible otherwise in the overcrowded reproductive clinic at which the study was based. Misrepresentation of researchers lessens the neutrality of the researcher and introduces bias in research. On the other hand it could enhance researcher – participant communication. To address this issue, researchers should communicate their role clearly to research participants [23].

### Gender, decision making and research

Women in our study were as willing as men to participate in research, except for a study involving sensitive questions. In contrast, significantly lower willingness to participate in research among women has been reported in literature for non HIV trials [35]. In a study involving microbicide use for HIV prevention in Thailand, only six percent of women reported a definite willingness to participate, while two thirds wanted time to think prior to making their decision. The necessity to consult with a spouse or partner was cited as a probable reason for the delay in decision making [36]. Similarly, most women in our study wanted time to confirm their decision, as the decision would be made by their spouse or family. Influence of spouse and family on decision making for participation in research has also been reported by an Egyptian study [37]. The low education and poor economic empowerment of many groups of women, in some parts of India [38] could influence autonomy as reported in our study.

The ethical principle of autonomy is compromised when decisions for potential participant's are made by others. Decision making is a complex phenomenon in many cultures [22]. Interfering with prevalent customs in order to ensure autonomy may lead to conflict within participants' family. Hence, informed consent procedures that encourage participatory decision-making within families should be developed to ensure ethical research practice. This is particularly relevant in the case of women participants who are less likely to make individual autonomous decisions in low-middle income settings.

Informed consent seeks to address two main moral concerns, i.e. autonomy and justice, for potential participants, while upholding the principles of beneficence and non-maleficence. When patients have a poor understanding of what research is or that their participation is voluntary the principles of informed consent are compromised. Uninformed participants could underestimate/have no comprehension of the risks of participation or of their rights as participants if they experienced an adverse event. A paternalistic doctor patient relationship compromises both autonomy and justice when participants consent to research because they think their physician has their best interests at hand. Autonomy is also compromised when participants misinterpret the purpose of research and try to fulfill their health needs when healthcare is a scarce resource [31]. The quality of life of participants in such situations could be compromised when time and money are lost due to participation. The principles of informed consent permit participants to drop out of research in due course of participation. The purpose is to provide ill/misinformed participants an opportunity to withdraw from research when their knowledge improves in due course of participation [39]. Poorly informed and obligated participants may also drop out of research either because their health needs are not fulfilled or because they are uncomfortable participating. This comes at a cost i.e. the power of research studies is affected when participants drop out of research. Additionally, uninformed participants with little education could misinterpret questions or provide researcher/physician desired responses, biasing the results.

### Methodological considerations

The study center is a non-profit, private tertiary level teaching hospital. As an academic institution with ongoing active research, patients may be better exposed to research than at other facilities. This environment could have influenced participant responses in our study. The generalizability of the results should therefore be considered in this light. Additionally, we did not include caste distribution, an indicator of healthcare equity in the Indian context, as a demographic variable. Also, willingness to participate assessed as a binary response, may have resulted in acquiescence by default [40]. A larger number of women in this study were unemployed and uneducated in comparison to men, thus affecting some responses where gender is known to play an important role. Overall, although the quantitative approach used in this study provides useful information, it has its limitations. A subsequent qualitative approach is therefore considered necessary to explore some responses in depth. Our study also did not address issues of monetary incentives that often influence consent. During the study, prior to assessing willingness to participate, we explained the meaning of research to all participants. Insufficient time to internalize and reflect on this concept, prior to stating their willingness to participate in research, could have positively influenced willingness to participate. It could also have raised awareness regarding research and minimized the influence of demographic variables on the outcome assessed.

## Conclusions

The finding in our study that 40% of the participants did not know what research meant and a third did not recognize that they could refuse participation implies that for a third of the patients, research participation could possibly be involuntary. Personal health benefits (self-interest) motivated most of the respondents to participate in research, followed by altruism. Though willingness to participate in research was not influenced by sex, women were less likely to make autonomous decisions and more likely to require time to decide regarding their participation. Researchers need to develop strategies that support contextual decision making processes in the Indian setting.

Uninformed participants could be easily coerced into participating in research. When this happens it could adversely affect not only the participants but also the research study. Therefore, effective communication by researchers to improve participant knowledge regarding research is necessary. Researchers should be sensitive to the fact that not all potential participants in low-middle and low-income settings know what research means or understand the implications of their participation. Hence research related knowledge may be improved through contextually suitable communication tools followed by reinforcement through one to one interactive sessions [12]. The exclusion of potential participants who fail to understand the concepts of research could be considered. This will ensure that the principles of justice and autonomy are adhered to. Participatory decision-making by the potential participants with their families should be encouraged based on the cultural context. Vulnerable populations like HIV infected individuals should be involved in research only when such research directly benefits them. These efforts will serve to uphold the principles of informed consent laid forth for the ethical conduct of research.
